# Differentiating Upper Extremity Necrotizing Soft Tissue Infection From Serious Cellulitis and Abscess

**DOI:** 10.7759/cureus.17806

**Published:** 2021-09-07

**Authors:** Landon E Cohen, Hyunwoo Kang, Kristen Sochol, Samuel A Cohen, Alidad Ghiassi, Milan Stevanovic, Rachel Lefebvre

**Affiliations:** 1 Orthopaedic Surgery, University of Southern California Keck School of Medicine, Los Angeles, USA; 2 Surgery, Stanford University School of Medicine, Palo Alto, USA

**Keywords:** necrotizing, soft tissue, cellulitis, abscess, infection

## Abstract

Introduction

Necrotizing soft tissue infection (NSTI) of the upper extremity (UE) is a rapidly progressing infection that requires early diagnosis and emergent treatment to decrease risks of loss of limb or life. Clinical presentation, particularly of early NSTI, can appear similar to serious cellulitis or abscess. The purpose of this study was to identify factors that are associated with NSTI rather than serious cellulitis and abscess to differentiate patients with similar clinical presentations.

Methods

This study uses a retrospective cohort design that compares patients ultimately diagnosed with UE NSTI versus those diagnosed with UE serious cellulitis or abscess. Cohorts were matched using the Laboratory Risk Indicators for Necrotizing Fasciitis (LRINEC) score in the setting of UE soft tissue infection. Laboratory values, vital signs, subjective symptoms, and social factors including substance abuse and domiciled status were recorded. Continuous variables were compared using the Mann-Whitney U test, whereas categorical variables were compared using the chi-squared test or the Fisher exact test (for expected values less than 5). A binary logistic regression for continuous and categorical variables was also performed. Significance was set at p<0.05. Univariate and multivariate analyses were performed.

Results

Multivariate statistical analysis and clinical interpretation of data identified four factors more associated with a diagnosis of NSTI than serious cellulitis or abscess: elevated lactate on hospital presentation, a patient-reported history of fever, male gender, and homelessness.

Conclusions

In patients with upper extremity infections, the clinical presentation of NSTI and serious cellulitis or abscess may appear similar. In this retrospective cohort of patients matched with LRINEC scores, elevated lactate, subjective fever, male gender, and homelessness were significantly associated with NSTI rather than serious cellulitis or abscess.

## Introduction

Necrotizing soft tissue infection (NSTI) is a rapidly advancing, necrotizing infection of the skin, subcutaneous tissue, and fascia [1]. Upper extremity (UE) NSTI is both a limb- and life-threatening condition [2]. Published mortality rates for UE NSTI range from 20% to 45%, with reviews and meta-analyses publishing overall mortality rates of about 20% [3-8]. UE NSTI requires emergent operative management including thorough debridement and intravenous antibiotics. Timely UE NSTI care is imperative; it has been shown that mortality rates increase steadily with each 24-hour period before the first operative debridement [5].

The first step in efficiently treating NSTI is diagnosing the condition [9]. Recognizing NSTI can be challenging, because it can occur in the absence of a known causative factor or portal of entry for bacteria [10]. Additionally, early symptoms of NSTI such as swelling, erythema, pain, and drainage from wounds are non-specific and can occur with many different types of infections [11]. Lack of specificity in clinical presentation often requires that multiple soft tissue infections - like serious cellulitis and abscess - with different treatments and prognoses be included in the differential [1]. Since the delay in NSTI diagnosis contributes to increased mortality, an accurate and efficient diagnosis of NSTI is imperative for successfully treating patients with NSTI [10].

In 2004, a diagnostic scoring system called the Laboratory Risk Indicators for Necrotizing Fasciitis (LRINEC) score was created for this purpose [12]. The goal of the LRINEC score was to create a “novel, simple, and objective scoring system” based on routine laboratory values that could help distinguish NSTI from other soft tissue infections [12]. In the years since, the literature examining LRINEC has shown the varied success of the LRINEC score. While the original authors describe a 92% positive predictive value and a 96% negative predictive value, more recent studies have found positive predictive values as low as 29% and some authors have questioned if LRINEC scores added any diagnostic value [12-17].

The goal of this study is to compare a matched retrospective cohort of patients with UE NSTI to those with serious cellulitis and abscess. Even if LRINEC scores cannot be reliably used to diagnose NSTI, they can be used to identify patients with similar laboratory findings on presentation. In our experience, this is often the very group of patients where the diagnosis of NSTI versus serious cellulitis or abscess is clinically challenging. Within these two groups of similar LRINEC scores, this study identifies factors associated with NSTI rather than serious cellulitis or abscess to help in the clinical differentiation between these diagnoses that require different treatments.

We hypothesized that certain laboratory findings, some within the LRINEC score, such as the white blood cell count (WBC), and some not accounted for by the LRINEC score, such as the lactate, may be associated with NSTI. We further hypothesized that vital signs at the emergency department presentation may differ between the two groups. Finally, we hypothesized that social conditions - in particular intravenous (IV) substance abuse and housing status - may be associated with NSTI versus serious cellulitis and abscess. 

## Materials and methods

All materials and methods in this study have been approved by the Institutional Review Board (IRB), and all necessary HIPAA consent has been received. The research meets all applicable standards with regard to the ethics of experimentation and research integrity. The paper has been submitted with full responsibility, following due ethical procedure, and there is no duplicate publication, fraud, plagiarism, or concern about animal or human experimentation.

Forty-two consecutive, surgically confirmed cases of UE NSTI from a single academic institution over a four-year period (2016-2019) were reviewed. Two patients were excluded: one with leukemia and lab abnormalities due to both leukemia and NSTI; and one who transferred to another hospital before NSTI work-up and care was complete. Thus, 40 patients were analyzed in the NSTI study. A control population of 40 consecutive cases of UE serious cellulitis and/or abscess were also reviewed. The control population was matched to the NSTI cohort through average LRINEC scores. Because the average LRINEC score for the NSTI group was expectedly elevated, eligibility for the control group included a LRINEC score of 3 or greater. The goal of this design was to focus on the cases where it is challenging to clinically differentiate between NSTI and cellulitis or abscess. All cases and controls were treated at a major urban, safety-net hospital.

For all included patients, variables collected for data analysis were grouped into one of three categories: lab values, vital signs at Emergency Department (ED) presentation, and qualitative data on symptoms and social factors.

Lab values collected for each patient included LRINEC score and its six components [C-reactive protein (CRP), white blood cell count (WBC), hemoglobin (Hb), sodium (Na), creatinine (Cr), and blood glucose], erythrocyte sedimentation rate (ESR), lactate, and international normalized ratio (INR). Vital signs at ED presentation that were collected for each patient include maximum temperature (Tmax), maximum heart rate (HRmax), maximum respiratory rate (RRmax), maximum systolic blood pressure (SBPmax), minimum systolic blood pressure (SBPmin), maximum diastolic blood pressure (DBPmax), minimum diastolic blood pressure (DBPmin), maximum mean arterial pressure (MAPmax), minimum mean arterial blood pressure (MAPmin), change in systolic blood pressure (dSBP), change in mean arterial pressure (dMAP), maximum pulse pressure (PPmax), and minimum pulse pressure (PPmin). Additional identifying information, including age and time to presentation, were also grouped into this category. The patient and infection characteristic variables recorded for each case include patient gender, laterality of injury (right or left), homelessness, patient provided history of recent intravenous substance use, subjective fever, and objective fever.

For our statistical analyses, all continuous variables were compared with Mann-Whitney U, whereas categorical variables were compared with chi-square test or Fisher exact test (for expected values less than 5). A binary logistic regression for continuous and categorical variables was also performed. Significance was set at p<0.05. Univariate and multivariate analysis was performed.

## Results

In analysis of the quantitative date: lab results and vital signs, univariate and multivariate analyses were performed. Univariate analysis of lab values identified statistically significant differences in two variables - WBC and lactate (Table 1). WBC values were significantly higher in the cohort of patients with NSTI (21.23 ± 9.94) compared to the cohort with cellulitis and abscess (15.19 ± 6.20) (p = 0.005). Lactate levels were significantly higher among NSTI patients (2.06 ± 1.43) than cellulitis/abscess patients (1.35 ± 0.66) (p = 0.002). Notably, other components of the LRINEC score, such as CRP, Hb, Na, Cr, and glucose were not different between the two groups. In the multivariate analysis of these laboratory variables, only elevated lactate remained statistically significant (OR = 4.696, p = 0.007). Univariate analysis of vital signs data identified statistically significant, but clinically insignificant, differences in three variables - SBP max, dSBP max, PPmax (Table 1). In the multivariate analysis, no differences in vital signs were found to be statistically significant.

**Table 1:** Lab values of control vs. necrotizing fasciitis cohorts.

	Control group, N = 65		Necrotizing fasciitis, N = 40		
Continuous variables	Mean ± standard deviation	Range	Mean ± standard deviation	Range	p-value
LIRNEC score	5.15 ± 2.20	(3-10)	6.09 ± 3.15	(0-12)	-
Age	49.18 ± 13.18	(26-91)	46.08 ± 13.83	(23-88)	0.167
Time to presentation (days)	5.69 ± 4.19	(0-14)	7.43 ± 8.82	(0.1-50)	0.669
Tmax (maximum temperature in ER)	37.52 ± 0.73	(36.1-39.5)	37.72 ± 0.91	(36.4-39.7)	0.457
HRmax (maximum heart rate in ER)	103.40 ± 18.19	(66-145)	111.50 ± 19.67	(73-149)	0.061
RRmax (maximum respiratory rate in ER)	23.00 ± 12.06	(18-94)	23.03 ± 5.93	(10-39)	0.255
SBPmax (maximum systolic blood pressure in ER)	149.52 ± 16.36	(118-194)	139.18 ± 20.76	(104-198)	0.018
DBPmax (maximum diastolic blood pressure in ER)	85.78 ± 13.96	(55-114)	85.75 ± 13.95	(46-117)	0.795
MAPmax (maximum mean arterial pressure in ER)	107.03 ± 13.36	(83-140)	103.56 ± 13.85	(77.3-129.3)	0.268
SBPmin (minimum systolic blood pressure in ER)	112.55 ± 18.24	(57-151)	111.35 ± 17.71	(73-144)	0.567
DBPmin (minimum diastolic blood pressure in ER)	67.60 ± 12.19	(35-96)	67.60 ± 12.46	(46-95)	0.973
MAPmin (minimum mean arterial pressure in ER)	82.58 ± 13.26	(58.3-112.3)	82.18 ± 13.50	(60-114.3)	0.736
dSBP (delta systolic blood pressure)	36.98 ± 15.35	(5-100)	27.83 ± 22.07	(0-80)	0.034
dMAP (delta mean arterial pressure)	24.44 ± 11.29	(3-43.3)	21.38 ± 15.78	(0-68.7)	0.222
WBC (white blood cells)	15.19 ± 6.20	(3.8-25.9)	21.23 ± 9.94	(6.8-61)	0.005
Hb (hemoglobin)	11.47 ± 2.83	(4.9-16.6)	12.05 ± 2.05	(7.3-15.9)	0.465
Na (sodium)	134.08 ± 5.62	(117-151)	134.93 ± 7.01	(120-163)	0.440
Cre (creatinine)	2.06 ± 1.43	(0.39-5.17)	1.34 ± 1.68	(0.47-9.15)	0.278
Glc (glucose)	215.75 ± 188.43	(83-1,005)	172.49 ± 152.14	(74-785)	0.057
CRP (C-reactive protein)	153.13 ± 93.50	(1.6-356.2)	211.14 ± 128.70	(12.6-559.7)	0.068
ESR (erythrocyte sedimentation rate)	62.35 ± 35.35	(15-115)	54.00 ± 28.71	(8-102)	0.407
Lactate	1.35 ± 0.66	(0.4-3.5)	2.06 ± 1.43	(0.5-8.7)	0.002
INR (international normalized ratio )	1.15 ± 0.13	(0.86-1.46)	1.24 ± 0.24	(0.95-2.26)	0.076
PPmax (maximum pulse pressure)	63.75 ± 13.55	(39-92)	53.43 ± 19.16	(20-92)	0.001
PPmin (minimum pulse pressure)	44.95 ± 12.38	(18-71)	43.75 ± 10.74	(25-68)	0.881

 

In the analysis of qualitative data, univariate analysis identified significant differences in four variables - homelessness, IVDU, subjective fever, and gender (Table 2). Patients with NSTI were significantly more likely to be undomiciled (67.5%) than patients with cellulitis or abscess (23.7%) (χ^2^ = 15.05, p < 0.001). Additionally, patients in the NSTI cohort were more likely to abuse IV drugs (65.0%) than patients in the cellulitis and abscess cohort (27.5%) (χ^2^ = 11.3, p = 0.001). Patients with NSTI diagnosis were more likely to have self-reported a subjective fever (50.0%) than patients with cellulitis or abscess (7.9%) (χ^2^ = 18.65, p < 0.001). In univariate analysis, patients with NSTI (70%) (χ^2^ = 5.94, p = 0.015) were less likely to be male than cellulitis and abscess patients (92.5%), although the majority of patients in both groups were male. In the multivariate analysis, subjective fever (OR = 13.731, p = 0.002), homelessness (OR = 13.844, p = 0.001), and gender (OR = 15.45, p = 0.017) all remained statistically significant (Table 3).

**Table 2:** Categorical variables for the control and necrotizing fasciitis cohorts.

	Total		Control group, N = 65		Necrotizing fasciitis, N = 40		χ^2^	p-value
Categorical variables	n = 80		n = 40		n = 40			
	n	%	n	%	n	%		
Gender								
Male	65	77.4%	37	92.5%	28	70.0%	6.65	0.01
Female	15	17.9%	3	7.5%	12	30.0%		
Laterality								
Right	51	60.7%	26	65.0%	25	62.5%	0.05	0.816
Left	29	34.5%	14	35.0%	15	37.5%		
Homeless								
Yes	36	42.9%	9	23.7%	27	67.5%	15.05	<0.001
No	42	50.0%	29	76.3%	13	32.5%		
IV drug use								
Yes	37	44.0%	11	27.5%	26	65.0%	11.31	0.001
No	43	51.2%	29	72.5%	14	35.0%		
Subjective fever								
Yes	23	27.4%	3	7.9%	20	50.0%	16.62	<0.001
No	55	65.5%	35	92.1%	20	50.0%		
Objective fever								
Yes	19	22.6%	6	15.0%	13	32.5%	3.38	0.066
No	61	72.6%	34	85.0%	27	67.5%		

**Table 3:** Binary logistic regression of all statistically significant variables. CI: Confidence Interval

							95% CI for EXP(B)	
Factor	B	SE	Wald	df	Sig.	Exp(B)	Lower	Upper
Gender	2.738	1.149	5.675	1.000	0.017	15.450	1.624	146.949
Subjective fever	2.620	0.865	9.181	1.000	0.002	13.731	0.013	0.396
Homeless	2.628	0.816	10.376	1.000	0.001	13.844	2.798	68.501
Lactate	1.547	0.571	7.336	1.000	0.007	4.696	1.533	14.380
Constant	-2.276	1.287	3.128	1.000	0.077	0.103	-	-

## Discussion

Early diagnosis and surgical debridement of UE NSTI is a critical part of treatment in patients with this limb and life-threatening condition. It is often challenging to definitively diagnose NSTI when the clinical picture is similar to serious cellulitis or abscess. By creating a matched group of control patients with UE cellulitis and abscess with, by design, similar LRINEC scores, this research identifies other observable clinical factors associated with NSTI rather than cellulitis or abscess.

Our results indicate that WBC and lactate levels differ between patients with NSTI and those with serious cellulitis and abscess. While the NSTI group and control group were initially matched through average LRINEC scores, WBC - a component of LRINEC - still differed significantly between the two groups. Other published studies have not yet linked elevated lactate to NSTI diagnosis, but the clinical use of monitoring lactate levels in critically ill patients is common [18]. Lactate is a quick, reliable predictor of morbidity and mortality [18,19]. Additionally, lactate monitoring has been used successfully in risk-stratification for critically ill patients [19]. Specifically in regards to NSTI infections, lactate monitoring has been used in multiple studies as a predictor of NSTI mortality rates [20,21]. Although patients with serious cellulitis and abscess may appear clinically similar at presentation to those with NSTI, the association of higher WBC and higher lactate levels indicate a higher risk to limb and life in patients with UE NSTI.

Our results did not find a statistically or clinically meaningful difference in vital signs on initial presentation between the serious cellulitis and abscess group and the NSTI group. Both groups, on average, were tachycardic on hospital presentation. The cause of tachycardia is likely multifactorial. Although it was not clinically or statistically different between the two groups, it is important to record in all patients who present with serious UE infection. In the literature, tachycardia is described as a symptom of NSTI and, in some studies, it has been linked to increased morality rate in patients being treated for NSTI [22-25]. Although SBP max, dSBP max, PPmax were found to be statistically significant in univariate analysis, the differences were not found to be statistically significant in multivariate analysis. Moreover, we do not find the differences in the values to be clinically significant.

Analysis of our data indicates that social factors, including housing status and IV drug abuse, are more associated with UE NSTI versus serious cellulitis or abscess of the UE. A strong association between homelessness and UE NSTI was found in both univariate and multivariate analysis. There is limited data in the current literature linking homelessness to NSTI infection. There are epidemiological studies describing outbreaks of Group A Streptococcal (GAS) infection - a cause of both NSTI and cellulitis/abscess -among a homeless population with an incidence of up to 53 times the domiciled population [24,26]. However, our study is the first that we know of to report a statistically significant association between homelessness and diagnoses of NSTI as opposed to other serious UE soft tissue infections. 

Though both study populations contained a majority of male patients, gender showed statistical significance in both univariate and multivariate analysis. The group of patients with NSTI was overwhelmingly male compared to the group of patients with serious cellulitis and abscesses. This finding is consistent with prior studies reporting higher rates of both cellulitis [27,28] and NSTI [29] in male patients. However, the clinical significance of this statistical finding is limited, because both groups with serious soft tissue UE infections show a significant association with male gender.

An additional statistically significant variable found in the analysis of our data is a history of subjective fevers. Although subjective fever was more associated with NSTI versus serious cellulitis and abscess, measured temperature and the presence of an objective fever in the emergency department were not significantly associated with one diagnostic group on either univariate or multivariate analysis. Subjective reports of symptoms have been linked to NSTI diagnosis previously, but this is the first association between UE NSTI and subjective fever [30]. 

There are several weaknesses of this study. Firstly, this is a single-center, retrospective study with a relatively small sample size that may not be generalizable to other patient populations. Our institution is an academic, large, urban, safety-net hospital and may have a baseline patient population different from other institutions. In our patient population, UE NSTI seems to disproportionately affect an under-researched and under-served population of individuals with a high rate of IV drug abuse and homelessness. Additionally, some metrics used in this study (such as subjective fever) were patient-reported and therefore we were not able to verify their accuracy. Finally, because NSTI is a time-sensitive diagnosis, one laboratory value should not delay proper treatment. As such, the results of our study should be interpreted with caution. Future studies to hone our early diagnosis of NSTI versus serious cellulitis and abscess should be prospective and, instead of using LRINEC scores to match a cohort, could identify patients on clinical presentation where the diagnosis of NSTI versus serious cellulitis and abscess is in question. 

## Conclusions

In conclusion, swift and reliable differentiation of UE NSTI from serious abscess and cellulitis is of vital clinical importance. This may lead to a limb or life-saving surgical intervention. It is often clinically challenging to accurately differentiate between these illnesses. In cases where the clinical and laboratory evaluation may not give a clear diagnosis, an elevated WBC, lactate, homelessness, and subjective fever should lead clinicians to an increased consideration of UE NSTI for their diagnosis.
